# Editorial: Applications and Methods in Genomic Networks

**DOI:** 10.3389/fgene.2022.936015

**Published:** 2022-06-08

**Authors:** Maud Fagny, Kimberly Glass, Marieke L. Kuijjer

**Affiliations:** ^1^ EcoAnthropology Lab, UMR 7206 CNRS/MNHN/Universite Paris Diderot, Muséum National d'Histoire Naturelle, Paris, France; ^2^ Université Paris-Saclay, INRAE, CNRS, AgroParisTech, GQE — Le Moulon, Gif-sur-Yvette, France; ^3^ Channing Division of Network Medicine, Brigham and Women’s Hospital, Boston, MA, United States; ^4^ Harvard Medical School, Boston, MA, United States; ^5^ Harvard Chan School of Public Health, Boston, MA, United States; ^6^ Centre for Molecular Medicine Norway (NCMM), Nordic EMBL Partnership, University of Oslo, Oslo, Norway; ^7^ Department of Pathology, Leiden University Medical Center, Leiden, Netherlands; ^8^ Leiden Center for Computational Oncology, Leiden University Medical Center, Leiden, Netherlands

**Keywords:** network inference, network modeling, biological network analysis, genomic networks, network applications

High-throughput technologies are generating large quantities of data. These data provide a snapshot of the molecular environment and can include transcriptomic, epigenomic, and genomic information. Network approaches are a powerful way to model the biological processes measured by these data. Over the past decade, network inference and reconstruction algorithms have been developed and applied in a variety of organisms and tissues to model interactions between genes and gene products in the cell. Network approaches hold great promise in facilitating our understanding of biological processes, as well as their relationship to health and disease. However, there are many challenges that impede translating ‘omics data into meaningful networks, and in leveraging networks effectively to gain new insights into biological mechanisms and/or impact patient outcomes. Networks derived from ‘omics data are often very large and therefore difficult to model, analyze, and interpret. The Research Topic on “Applications and Methods in Genomic Networks” covers several areas—from discussions about how to handle data prior to network modeling, to the presentation of innovative and novel methods for biological network inference and analysis, to how to make the results available to and usable by the genomic network community, to applications illustrating the impact of network approaches in a wide range of research fields in biology and medicine.

First, this collection contains articles tackling a wide variety of issues related to genomic network inference and analysis. Cuesta-Astroz et al. propose an approach to improve data filtering, reduce noise, and increase signal in biological networks. An important challenge in the field of network biology is to develop inference methods that retrieve actual regulatory relationships while limiting the number of false positives, and that are not overly sensitive to noise. Random forest-based methods are efficient at detecting true regulatory relationships, but create a high proportion of false positives and thus, pruning networks built with these approaches is necessary to avoid spurious regulatory relationships. To solve this issue, Kimura et al. built a pipeline combining an efficient random forest-based network inference method with a series of feature selection methods, which significantly improved the quality of the inferred network. Network inference methods should also lead to consistent results across datasets obtained from the same biological condition. This is particularly important for data-driven approaches applied to single-cell datasets, which are known to have a high level of inherent noise. Kang et al. propose a blueprint to benchmark network inference approaches in this context, that includes most genes present in the network, taking into account both their presence in the network and the weight of their relationships, and assesses the biological soundness of the inferred networks by comparing them to gold-standard regulatory relationships extracted from public databases.

Once researchers have properly filtered their data, inferred the networks, and filtered out spurious connections, the networks are ready for analysis. A common way of making sense of genomic networks, which often include thousands of nodes and many more edges, is to look for modules or communities, *i.e.* groups of nodes that are enriched for links to each other relative to other parts of the network. Identifying network communities, and comparing them across conditions, are two ways of identifying condition-specific regulatory relationships and extracting new biological knowledge from a network. However, finding modules within a network is an NP-hard problem, meaning that existing approaches for large networks approximate the best community structure. This raises the question of the robustness of the network structure detected and its biological interpretability. Several papers in this collection address this issue from different angles. A mini-review by Calderer and Kuijjer compares different algorithms to infer modules from bipartite networks and proposes different scores aiming at assessing the quality of each method. Three other articles focus on the comparison of networks between conditions. In a perspective piece, Weighill et al., highlight the promise of using a weighted gene degree, or “gene targeting score,” toglobally compare networks inferred from data representing different conditions, in order to identify key regulatory processes in disease. Lim et al. developed Constrained Random Alteration of Network Edges (*CRANE*), a new algorithm to identify robust disease-related regulatory modules. Finally, Arbet et al. share a new algorithm aimed at identifying differentially co-expressed modules and propose an R implementation, *discoMod*. This tool tests whether connections between co-expressed genes differ between conditions, and allows the user to assess how regulatory relationships within a module vary between conditions.

Finally, two papers tackled an important issue in the genomic network field: how to disseminate genomic network results and make them usable by the broader scientific community. Yang et al. built a web platform that hosts co-expression network results from *Gastrodia elata,* an important herb in traditional Chinese medicine. The platform gives access to a series of tools that facilitate result-browsing and allows the user to perform functional analysis of genes. Garcia-Ruiz et al. propose CoExp, a web platform that allows researchers to manipulate, compare, and analyze 109 co-expression networks. Importantly, the types of web tools presented here, based on open data and widely used programming languages and softwares, can be emulated and applied to a wide range of topics and organisms.

Rapidly developing research on how to best infer the genomic networks has led to the publication of algorithms and software that are crucial tools in systems biology. Many of these tools focus on unraveling the biological networks involved in regulating gene expression at the level of a cell, tissue, or organism. The application of these tools is leading to crucial discoveries in fields as diverse as Alzheimer’s disease (Brabec et al.), the control of mitochondrial gene expression in *D. melanogaster* (Cuesta-Astroz et al.), the response to abiotic stress in rice (Sharma et al.), and cancer.

In this collection, five articles from the Computational Genomics Division of the National Institute of Genomic Medicine in Mexico City use mutual information approaches to explore gene co-expression networks associated with diverse cancer stages. Zamora-Fuentes et al. analyzed both gene expression and co-expression modeled on data obtained from different stages of clear cell renal carcinoma, and found substantial differences in network topology across cancer stages, with a loss of interchromosomal (*trans*) interactions compared to control networks. A similar observation was made in lung cancer by (Andonegui-Elguera et al.). Guarcia-Cortés et al. further analyzed differences in inter- and intrachromosomal (*cis*) interactions in the luminal A subtype of breast cancer. They found that *cis*-communities were enriched in copy number deletions, representing a potential mechanism of strengthened *cis*-co-expression and loss of *trans*-co-expression in cancer. Ochoa et al. also focused on breast cancer, modeling multilayer networks based on various types of omics data and identifying potential regulatory patterns of breast cancer subtype expression. Finally, through combining gene-microbiome networks with co-expression networks in colon cancer Uriart-Navarrete et al. characterized discriminating features between early and late stage cancer.

In summary, this Research Topic presents a wide variety of novel methods for network pre-processing, modeling, benchmarking, and comparison, as well as applications of network analysis to integrate different data layers, study the control of gene expression in model organisms, as well as investigate altered associations and network properties in response to environmental triggers and disease. We believe that, together, these articles form a strong basis for discussions and future projects supporting novel method development in genomic network science, as well as future applications of large-scale network modeling in biology.

